# Functional status of women with and without potentially life‐threatening maternal conditions after 6 months postpartum: A cohort study

**DOI:** 10.1002/ijgo.70905

**Published:** 2026-03-13

**Authors:** Fitiwi Tinsae Baykemagn, Girmatsion Fisseha Abreha, Yibrah Berhe Zelelow, Alemayehu Bayray Kahsay

**Affiliations:** ^1^ College of Medicine and Health Sciences Adigrat University Adigrat Tigray Ethiopia; ^2^ School of Public Health, College of Health Sciences Mekelle University Mekelle Tigray Ethiopia; ^3^ Department of Obstetrics and Gynecology, School of Medicine Mekelle University Mekelle Tigray Ethiopia

**Keywords:** disability, functioning, severe maternal morbidity, WHODAS 2.0

## Abstract

**Objective:**

The aim of this study was to evaluate the impact of potentially life‐threatening maternal conditions (PLTCs) on functional disability at 6 months postpartum.

**Methods:**

This prospective cohort study was done at 10 hospitals in Tigray, northern Ethiopia. A total of 1027 postpartum women (341 with PLTCs and 686 without) were enrolled into the exposed and the unexposed groups, respectively. Disability status was assessed using the WHO Disability Assessment Schedule 2.0 (WHODAS 2.0). Data were collected through interviews and card reviews. Statistical analyses were performed using Pearson's chi‐square and Mann–Whitney for bivariate analyses. Due to the non‐parametric distribution of the outcome and the covariate (baseline WHODAS 2.0) data, we used non‐parametric analysis of covariance (ANCOVA) to assess WHODAS 2.0 score differences between the groups at 6 months postpartum, adjusting for baseline WHODAS 2.0 scores and other covariates.

**Results:**

Among 1027 participants, 997 (97%) completed the 6‐month follow‐up. Women with PLTCs had significantly higher median WHODAS 2.0 scores (25.0 vs. 9.4, *P* < 0.001; effect size = 0.34) and increased disability levels across all domains (*P* < 0.001). The non‐parametric ANCOVA showed that PLTC had a significant independent effect (partial eta‐squared (η^2^p) = 0.043, *P* < 0.001) after adjusting for confounders (demographic and clinical variables).

**Conclusion:**

Women who experienced PLTCs had significantly higher functional disability than those who did not at 6 months postpartum. The effects of PLTCs extend beyond the conventional 6‐week postpartum period. Establishing new mechanisms for long‐term maternal health follow‐up is essential to address ongoing functional disability.

**Abbreviations**
η^2^p: partial eta‐squared
ANC: antenatal care
ANCOVA: analysis of covariance
CS: cesarean section
IQR: interquartile range
PLTCs: potentially life‐threatening maternal conditions
SD: standard deviation
SDG: sustainable development goals
SVD: spontaneous vaginal delivery
WHODAS 2.0: World Health Organization disability assessment schedule II

## 1 INTRODUCTION

Globally, over 700 women die from preventable causes related to pregnancy and childbirth complications every day. However, the burden of morbidity is far greater, with more than 20 women surviving with potentially long‐lasting consequences for every maternal death.^1^ Severe maternal morbidity includes potentially life‐threatening maternal conditions (PLTCs) during pregnancy, labor and delivery, or the postpartum period.^2^ PLTCs includes hemorrhage, hypertension, severe management indicators, and other systemic conditions that threaten immediate survival. They also have the potential to cause long‐term disability.^3^ Globally, about 7% of pregnant women experience PLTCs, predominantly in low‐income countries.^4^ Disability is body impairment in function or structure, activity limitation, and participation restriction, which is a key determinant of postpartum recovery and quality of life.^5^ The WHO Disability Assessment Schedule 2.0 (WHODAS 2.0) assesses functioning and disability in six domains: participation, mobility, self‐care, getting along, life activities, and cognition.^6^

The existing literature indicates that PLTC is associated with adverse postpartum outcomes, including elevated functional disability levels compared to women without PLTCs.^7^, ^8^, ^9^ A study applying the short form‐12 WHODAS 2.0 has revealed significant relationships between PLTCs and functional disability,^10^ while other studies point out clinical outcomes like mortality and readmission.^8^, ^11^, ^12^ These results highlight the broader impacts of severe maternal morbidity on women's lives, encompassing difficulties with social engagement, newborn care, and returning to work.^3^

Despite these findings, there are still important unanswered questions about the long‐term functional impact of PLTCs, which this study attempts to address. Previous studies on PLTCs are limited by retrospective designs, small sample sizes, short follow‐up periods (mainly up to 6 weeks postpartum), lack of baseline disability data, and inadequate control for confounders. Consequently, there is insufficient evidence on the long‐term (6‐month) functional impact of PLTCs on postpartum disability and recovery.^9^, ^13^, ^14^, ^15^

This prospective cohort study overcomes these limitations by evaluating the long‐term impact of PLTCs on postpartum functioning and disability at 6 months. Conducted in a large cohort from a high‐burden, low‐resource setting, the study incorporates baseline disability assessment and uses non‐parametric analysis of covariance (ANCOVA) to adjust for confounders and account for non‐normal distributions. This study extends follow‐up to 6 months postpartum, isolates PLTCs effects, and applies robust methods, including a prospective design, a large sample size, the comprehensive use of the WHODAS 2.0, and rigorous confounder control. The study provides new, policy‐relevant evidence to inform extended postpartum care, disability screening, and rehabilitation strategies, thereby supporting SDG 3 targets for women's health.^16^ Therefore, the aim of this study was to evaluate the long‐term impact of PLTCs on functional disability at 6 months postpartum.

## 2 MATERIALS AND METHODS

### 2.1 Study design and area

A prospective cohort study design was conducted in the Tigray region, northern Ethiopia. Tigray region has two referral hospitals and 14 general hospitals, of which all 10 hospitals (the two referral hospitals and eight general hospitals) providing comprehensive emergency obstetric and newborn care (CEmONC) services for the management of PLTCs were selected. Six general hospitals were excluded due to security concerns (*n* = 4) and lack of capacity for comprehensive PLTCs management (*n* = 2).

### 2.2 Study population

**FIGURE 1:**
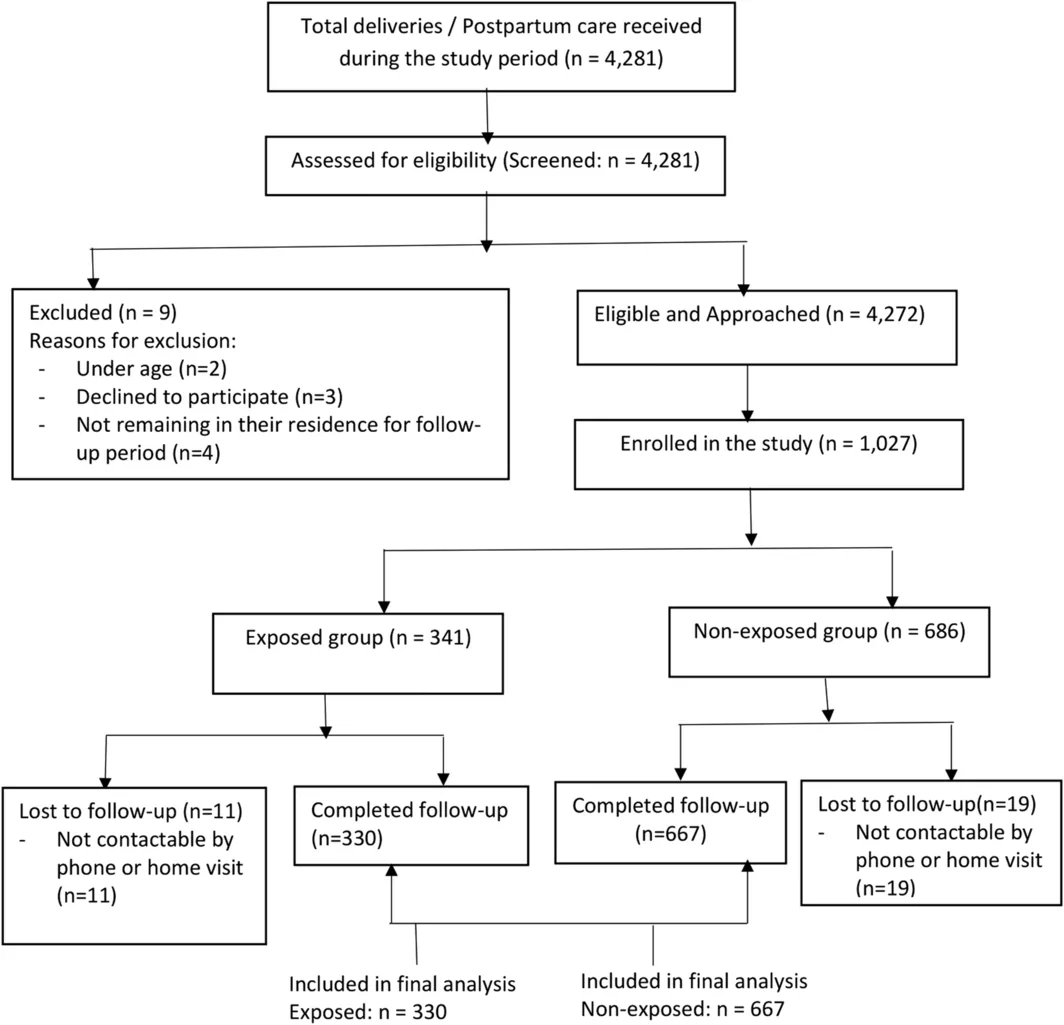
Flow chart of women participating in the study.

Eligibility criteria: Women were eligible if they were admitted to the selected hospitals for delivery or postpartum care between January 21 and April 20, 2024, and were at least 18 years old. All participants were then prospectively followed for 6 months postpartum, with final assessments completed between July 21 and October 20, 2024. The exposed group consisted of women who experienced at least one PLTC as defined by WHO criteria during pregnancy, childbirth, or within 42 days postpartum.^2^ The non‐exposed group consisted of women who did not experience any PLTC during pregnancy, childbirth, or within 42 days postpartum. Grouping (exposed vs. non‐exposed) was initially determined at enrollment based on a clinical assessment confirmed by an obstetrician/gynecologist. The exposed group included women diagnosed with a PLTC according to WHO criteria, while the non‐exposed group initially comprised women who did not have any PLTC at enrollment and who delivered at the same facility. However, during follow‐up, any woman in the non‐exposed group who subsequently developed PLTC criteria within the first 42 days postpartum was reclassified into the exposed group. Exclusion criteria (applied to both groups): Age less than 18 years due to ethical issues related to informed consent and when the mother cannot stay in her place of residence until the end of the study follow‐up period (6 months) to minimize lost to follow‐up. All women receiving delivery or postpartum care during the study period (*n* = 4281) were screened for eligibility (Figure 1).

### 2.3 Sample size and sampling technique

Sample size was calculated using Stata software (version 17) with the “power twomeans” command to detect the difference between two independent means. The required sample size per group was derived using the following expression:

$$n_{1}=\frac{\left(\sigma_{1}^{2}+\frac{\sigma_{2}^{2}}{k}\right)\cdot {\left(z_{1-\frac{\alpha }{2}}+z_{1-\beta }\right)}^{2}}{\Delta^{2}}$$

where: *n*_1_: the sample size for the exposed group, *n*_2_: the sample size for the non‐exposed group (*n*_2_ = *n*_1_*k). $\sigma_{1}$: standard deviations of the exposed group. $\sigma_{2}$: standard deviations of the non‐exposed group. *Z*₁₋*α*/₂: Significance level. *Z*₁₋*β*: power. Δ the difference in means. k: the allocation ratio (non‐exposed: exposed).

The sample size calculation was performed with an alpha value of 0.05, a power of 90%, a 1:2 allocation ratio (exposed to non‐exposed groups), and mean (SD) of the WHODAS scores from a previous study of 19.0 (16.1) for the exposed group and 15.7 (14.4) for the non‐exposed group.^7^ After adding a 5% non‐response rate, the required sample size was 1027 (341 in the exposed group and 686 in the non‐exposed group).

Proportional sample size allocation was then applied to each hospital based on the previous year's delivery volume. During the recruitment period, a total of 4281 women received delivery or postpartum care at the participating hospitals. All women diagnosed with PLTC (the exposed group) were consecutively enrolled until the required sample size was reached. For each enrolled PLTC case, two women without PLTC (the non‐exposed group) who delivered in the same hospital on the same day were randomly selected. The matching was based on facility and time to control for variations in quality of care, resources, and timing. We did not perform individual‐level matching on variables like gestational age, parity, or socioeconomic status to avoid over‐matching and loss of generalizability. Instead, potential confounding from these unmatched factors was addressed through statistical adjustment: age, parity, socioeconomic status, and other relevant covariates were included in the final non‐parametric ANCOVA model.

### 2.4 Data collection techniques and tools

The patients interview and records review questionnaire consisted of the following main sections: the sociodemographic characteristics, medical and obstetric history, PLTCs diagnosis criteria, and WHODAS 2.0 tool. The identification of the women with PLTCs was assessed as per the WHO criteria for PLTCs, as described elsewhere.^17^ The data were collected by midwifery professionals who are experienced in data collection and clinical practices. For every potential participant who agreed to take part, medical records were reviewed, data related to sociodemographic features and reproductive history were retrieved, and phone numbers were gathered. Participants were followed prospectively for 6 months postpartum. At baseline, all assessments were conducted via in‐person interviews combined with record review at the health facility prior to discharge. At the 6‐week postpartum follow‐up, all participants were initially contacted via telephone to assess ongoing maternal complications or health status changes. At the 6‐month follow‐up, of the 997 women who completed the assessment, 899 (90.2%) were interviewed in person and 98 (9.8%) by telephone. Both modes adhered to the identical WHODAS 2.0 interview protocol, in accordance with official WHO guidelines, which state that the instrument can be administered in person or over the telephone using standard general interview techniques.^5^ Recent validation studies have demonstrated very high agreement between face‐to‐face and telephone administration of the 36‐item WHODAS 2.0, supporting that telephone mode does not meaningfully compromise validity or scores in this context.^18^ To minimize loss to follow‐up, multiple contact details (the participant's phone number and that of a close relative) were collected at baseline. Participants received reminder calls at 6 weeks postpartum and again before the 6‐month follow‐up assessment to maintain engagement and schedule the final interview. Additional retention strategies included up to three reminder calls and, when necessary, in‐person visits assisted by community health workers for hard‐to‐reach participants. Loss to follow‐up was defined as failure to complete the 6‐month assessment despite these efforts.

To assure data quality, training was given for supervisors and data collectors for two days on medical record reviewing, interviewing techniques, informed consent for participants, and confidentiality of the data. Feedback from the pretest was incorporated into the tool accordingly. The data consistency and completeness were checked by supervisors. Additionally, the WHODAS 2.0 was administered in the local Tigrigna language using a version formally adapted and validated for this study per WHO guidelines. The adaptation process included forward translation, expert review, back‐translation, and reconciliation. The tool demonstrated strong content validity (scale‐level content validity index = 0.96). Psychometric validation among PLTCs (*n* = 121) confirmed excellent internal consistency (Cronbach's *α* > 0.95) and strong convergent validity (*r* = 0.61–0.79), establishing its reliability and validity for this context.^19^

### 2.5 Measures

Potentially life‐threatening maternal condition is operationally defined as a woman diagnosed with any of the following WHO identification criteria or conditions: hemorrhagic disorders (placenta abruptio, ruptured uterus, ectopic pregnancy, postpartum hemorrhage, and placenta accreta/percreta); hypertensive disorders (severe pre‐eclampsia, eclampsia, severe hypertension, hypertensive encephalopathy, and HELLP syndrome), other systemic disorders (endometritis, pulmonary edema, respiratory failure, thrombocytopenia <100 000/mm^3^, sepsis, seizures, thyroid crisis, and shock); and severe complication management indicators (blood transfusion, central venous access, hysterectomy, prolonged hospital stay (>7 postpartum days), admission to the intensive care unit (ICU), non‐anesthetic intubation, return to operating room, and surgical intervention), as described elsewhere.^17^ Diagnosis of PLTC was performed by an experienced obstetrician‐gynecologist according to the WHO criteria, which incorporate clinical signs, laboratory findings, and required interventions.^2^

Functional disability is defined according to the WHO's International Classification of Functioning, Disability and Health (ICF) description.^20^ Functional disability was assessed using the overall score of the 36‐item WHODAS 2.0. Overall standardized scores ranged from 0 to 100. Each item's degree of disability is assessed using a five‐point ordinal scale, with 0 (no difficulty) and 4 (cannot perform the activity), as described elsewhere.^5^

### 2.6 Statistical analysis

All data analyses were performed using SPSS version 24. Descriptive statistics are presented as frequencies and percentages for categorical variables and as medians (md) with interquartile ranges (IQR) for continuous variables, based on the data distribution. A wealth index was computed using principal component analysis (PCA) of household asset and amenity variables (e.g., ownership of durable goods, housing characteristics). The first principal component, which explains the maximum variance, was used to categorize participants into wealth tertiles (poor, middle, rich). Data were collected electronically via KoBoToolbox, which incorporated mandatory fields, automated validation checks, and skip‐logic programming to ensure data completeness at entry. As a result, no item‐level missing data occurred for any baseline or follow‐up variables among participants who completed both assessments. However, 30 participants (2.9% of the total sample of 1027) were lost to follow‐up and did not provide 6‐month WHODAS 2.0 scores (the primary outcome). These individuals were excluded from the primary analysis, resulting in a complete‐case analysis (listwise deletion). This approach was deemed appropriate because: the proportion of loss to follow‐up was low and the primary outcome was assessed at a 6‐month only. Potential non‐response bias was assessed by comparing baseline characteristics between participants retained and those lost to follow‐up (*P* > 0.14), showed no significant difference.

Differences between the exposed and non‐exposed groups for categorical variables were tested using the Pearson's chi‐squared, while continuous variables were compared using the Mann–Whitney test. Normality test of the WHODAS 2.0 scores (total and domain‐specific) was assessed using the Shapiro–Wilk test, which indicated non‐normal distribution (*P* < 0.05 for all). Statistical significance was set at *P* < 0.05 (two‐tailed). Initially, the difference in WHODAS 2.0 scores between the two groups at 6 months postpartum was examined using the Mann–Whitney test. Effect sizes for these comparisons were calculated using Cohen's *r* for Mann–Whitney *U* tests (interpreted as small = 0.1–0.3, medium = 0.3–0.5, and large >0.5).^21^

To adjust for pre‐existing differences in disability using baseline WHODAS 2.0 scores as a covariate, we planned to use an analysis of covariance (ANCOVA). However, due to the non‐normal distribution of WHODAS 2.0 scores (confirmed by Shapiro–Wilk tests, *P* < 0.05), a non‐parametric analysis of covariance (ANCOVA) was ultimately used instead of a parametric ANCOVA for each WHODAS 2.0 domain and the total scale. The procedure involved rank‐transforming the outcome (6‐month WHODAS scores) and covariate (baseline WHODAS scores), then performing a standard ANCOVA on the ranks, adjusting for confounders (e.g., age, residence, parity, education, wealth index, mode of delivery, and comorbidities) identified via bivariate analysis (*P* < 0.05). This test was used to compare the WHODAS 2.0 scores of exposed and non‐exposed groups at 6 months after controlling for baseline disability scores, as detailed in the instructions.^22^, ^23^ This analysis used partial eta‐squared (η^2^p) to measure effect size, quantifying the variance in the outcome explained by the group difference (PLTC vs. no PLTC) after controlling for covariates. Effects were interpreted as small (η^2^p = 0.01–0.06), medium (0.06–0.14), or large (≥0.14).^24^ Covariates were selected based on bivariate associations (*P* < 0.05) with the outcome and included baseline WHODAS 2.0 scores (the primary covariate) plus demographic (e.g., age, education, wealth index) and clinical variables (parity, mode of delivery, history of chronic hypertension).

To assess multicollinearity among the covariates included in the multivariable model, we calculated the variance inflation factor (VIF). All VIF values were less than 2 (below common thresholds of 5 or 10 that indicate problematic multicollinearity).^25^ This confirms that multicollinearity was not an issue in our analysis. Data on pre‐existing medical conditions were collected, including chronic hypertension, pre‐gestational diabetes, retroviral infection, sickle cell disease, autoimmune disorders, asthma, and thyroid disease. Due to very low frequencies of most conditions (except chronic hypertension), only chronic hypertension was included as a covariate in the multivariable analysis.

Ethical approval was obtained from the College of Health Sciences, Mekelle University; with the reference number IRB (MU‐IRB 2129/2023). Informed verbal consent was obtained before data collection started after a full explanation of the ethical principles for medical research involving human subjects (in accordance with the principles of the Declaration of Helsinki). Verbal re‐consent was obtained before each follow‐up interview (in‐person or telephone) using a standardized script that restated the study purpose, voluntary participation, confidentiality, and right to withdraw.

## 3 RESULTS

A total of 1027 women (341 women with PLTCs and 686 women without PLTCs) agreed to participate and were interviewed at discharge during the postpartum period. The response rates for completed data over the 6‐month period were 330 out of 341 (96.77%) for women with PLTCs and 667 out of 686 (97.23%) for women without PLTCs. We performed a non‐response bias analysis based on sociodemographic characteristics and obstetric history to assess the differences between respondents (997, or 97.08%) and non‐respondents (30, or 2.92%), thereby justifying the representativeness of the respondents used for evaluation. Generally, the sociodemographic variables, obstetric/gynecologic history, and WHODAS‐2 scores of non‐respondents were quite similar to those of the respondents (*P* > 0.14). At 6 months postpartum, 899 women (90.20%) completed the WHODAS 2.0 interview in person at the hospital or their home, while 98 (9.80%) were interviewed by telephone after verbal re‐consent.

### 3.1 Sociodemographic and clinical features of participants

Both groups, women with and without PLTCs, had a mean age of 28 years. Significant differences were found in marital status, with 96.4% of women with PLTCs having partners and 99.1% of women without PLTCs having partners (*P* = 0.005). Those with PLTCs were more likely to live in rural areas (31.2%) compared to 20.5% of women without PLTCs, with a significant *P* value (<0.001) (Table 1).

**TABLE 1:** Sociodemographic and clinical features of participants at immediate postpartum period.

Characteristics	Categories	PLTCs *n* (%)	Non‐PLTCs *n* (%)	Total *n* (%)	*P* value^a^
Age (years)	Mean (SD)	28.90 (6.26)	28.04 (5.36)	28.33 (5.68)	0.064
Marital status	Without partner	12 (3.6)	6 (0.9)	18 (1.80)	0.005
	With partner	318 (96.4)	661 (99.1)	979 (98.20)	
Place of residence	Rural	103 (31.2)	137 (20.5)	240 (24.10)	<0.001
	Urban	227 (68.8)	530 (79.5)	757 (75.90)	
Education level	No formal education	47 (14.2)	53 (7.9)	100 (10.0)	0.001
	Primary	50 (15.2)	69 (10.3)	119 (11.9)	
	Secondary	151 (45.8)	340 (51.0)	491 (49.2)	
	College and above	82 (24.8)	205 (30.7)	287 (28.8)	
Wealth index	Poor	134 (41.0)	198 (29.8)	332 (33.5)	0.001
	Middle	88 (26.9)	233 (35.0)	321 (32.4)	
	Rich	105 (32.1)	234 (35.2)	339 (34.2)	
Maternal occupation (main)	Housewife	239 (72.4)	436 (65.4)	675 (67.7)	0.011
	Government employee	55 (16.7)	167 (25.0)	222 (22.3)	
	Merchant	36 (10.9)	64 (9.6)	100 (10.0)	
Partner education	No formal education	37 (11.2)	45 (6.7)	82 (8.2)	<0.001
	Primary	61 (18.5)	63 (9.4)	124 (12.4)	
	Secondary	138 (41.8)	291 (43.6)	429 (43.0)	
	College and above	94 (28.5)	268 (40.2)	362 (36.3)	
Alcohol intake during pregnancy	Yes	265 (80.3)	474 (71.1)	739 (74.1)	0.002
	No	65 (19.7)	193 (28.9)	258 (25.9)	
Having history of still birth	Yes	67 (20.3)	34 (5.1)	101 (10.1)	<0.001
	No	263 (79.7)	633 (94.9)	896 (89.9)	
Parity	Median (IQR)	3 (2)	3 (2)	3 (2)	0.470
Type of pregnancy	Single	307 (93.0)	654 (98.1)	961 (96.4)	<0.001
	Twin	23 (7.0)	13 (1.9)	36 (3.6)	
Having chronic hypertension	Yes	43 (13.0)	6 (0.90)	49 (4.90)	<0.001
	No	287 (87.0)	661 (99.10)	948 (95.10)	
Had malaria during pregnancy	Yes	10 (3.0)	3 (0.40)	13 (1.30)	0.001
	No	320 (97.0)	664 (99.60)	984 (98.70)	
Timely ANC booking and completed visits	Yes	108 (32.7)	166 (24.9)	274 (27.5)	0.009
	No	222 (67.3)	501 (75.10)	723 (72.5)	
Iron foliate intake and compliance	With compliance	163 (49.4)	382 (57.30)	545 (54.7)	<0.001
	Never taken	45 (13.6)	41 (6.10)	86 (8.6)	
	Without compliance	122 (37.0)	244 (33.6)	366 (36.7)	
Type of labor onset	Spontaneous	206 (62.4)	598 (89.7)	804 (80.6)	<0.001
	Induced	84 (25.5)	33 (4.9)	117 (11.7)	
	Elective	40 (12.1)	36 (5.4)	76 (7.6)	
Mode of delivery	SVD	143 (43.3)	474 (71.1)	617 (61.9)	<0.001
	Instrument	35 (10.6)	61 (9.1)	96 (9.6)	
	CS	152 (46.1)	132 (19.8)	284 (28.5)	

Abbreviations: ANC, antenatal care; CS, cesarean section; IQR, interquartile range; PLTCs, potentially life‐threatening maternal conditions; SD, standard deviation; SVD, spontaneous vaginal delivery.

^a^ Chi‐square or Mann–Whitney U test.

Our analysis indicated that there was a significant difference in WHODAS 2.0 scores among the various categories of sociodemographic characteristics and clinical histories. Women aged ≥35 years had higher scores than those aged 18–34 years (23.69 vs. 18.69, *P* = 0.005). Women in rural residences had a higher score compared to urban residences (27.42 vs. 17.10, *P* < 0.001). Maternal education had an effect on the mean scores; women with no formal education attained a mean score of 23.86 (*P* = 0.021). Mothers with a history of stillbirth were associated with higher mean scores (31.05, *P* < 0.001) (Table 2).

**TABLE 2:** WHODAS 2.0 scores by categories of sociodemographic and maternal characteristics.

Characteristics	Categories	Number of women	WHODAS 2.0 score		*P* value^a^
			Mean ± SD	Median (range)	
Health status	PLTCs	330	30.62 (24.98)	25.00 (0.0–91.19)	<0.001
	No PLTCs	667	14.10 (15.48)	9.38 (0.0–93.75)	
Age	18–34 years	821	18.69 (20.07)	12.50 (0.0–93.75)	0.005
	≥35 years	176	23.69 (22.81)	15.63 (0.0–85.16)	
Marital status	With partner	979	19.51 (20.65)	13.28 (0.0–93.75)	0.377
	Without partner	18	22.44 (21.59)	12.50 (0.0–86.71)	
Residence	Urban	757	17.08 (18.38)	11.71 (0.0–86.71)	<0.001
	Rural	240	27.42 (25.08)	17.58 (0.0–93.75)	
Educational level	No formal education	100	23.86 (25.22)	15.63 (0.0–93.75)	0.021^b^
	Primary education	119	22.39 (20.10)	17.19 (0.0–71.10)	
	Secondary education	491	18.36 (20.54)	12.50 (0.0–92.19)	
	College and above	287	18.97 (19.09)	14.06 (0.0–86.72)	
Socioeconomic level (wealth index)	Poor	332	22.92 (23.85)	16.80 (0.0–93.75)	0.273
	Middle	321	17.22 (17.34)	11.72 (0.0–85.16)	
	Rich	339	18.13 (19.32)	13.28 (0.0–84.38)	
Occupation (main)	Housewife	675	19.06 (21.14)	11.72 (0.0–93.75)	0.098
	Government employee	222	20.44 (19.20)	14.84 (0.0–78.13)	
	Merchant	100	21.06 (20.57)	14.06 (0.0–86.72)	
Partner education	No formal education	82	21.83 (23.67)	10.16 (0.0–93.75)	<0.001^b^
	Primary education	124	28.21 (24.53)	17.97 (0.0–86.72)	
	Secondary education	429	19.19 (20.37)	14.06 (0.0–92.19)	
	College and above	362	16.55 (17.86)	11.33 (0.0–86.72)	
Alcohol intake during pregnancy	Yes	739	19.45 (19.93)	13.96 (0.0–93.75)	0.589
	No	258	19.91 (22.65)	12.50 (0.0–92.19)	
Previous still birth	Yes	101	31.05 (23.65)	25.00 (0.0–86.73)	<0.001
	No	896	18.28 (19.90)	12.50 (0.0–93.75)	
Parity	≤4	882	18.94 (20.31)	12.50 (0.0–93.75)	0.007
	≥5	115	24.38 (22.68)	24.38 (0.0–81.25)	
Type of gestation	Single	961	19.64 (20.85)	13.28 (0.0–93.75)	0.762
	Twin	36	17.58 (14.76)	17.19 (0.0–67.19)	
Having chronic hypertension	Yes	49	40.35 (26.90)	42.19 (0.0–93.75)	<0.001
	No	948	18.50 (19.72)	12.50 (0.0–92.19)	
Labor onset	Elective	76	25.57 (23.93)	17.97 (0.0–84.38)	0.018^b^
	Induced	117	22.92 (21.21)	14.84 (0.0–76.56)	
	Spontaneous	804	18.52 (20.12)	12.50 (0.0–93.75)	
Delivery mode	SVD	617	16.32 (19.16)	8.59 (0.0–85.16)	<0.001^b^
	Instrumental	96	22.55 (17.57)	19.53 (0.0–75.00)	
	CS	284	25.63 (23.16)	18.75 (0.0–93.75)	
Iron folate intake and compliance	With compliance	545	15.49 (18.23)	8.59 (0.0–92.19)	<0.001^b^
	Never taken	86	28.52 (24.97)	19.92 (0.0–93.75)	
	Without compliance	366	23.54 (21.55)	16.40 (0.0–86.72)	
Had malaria during pregnancy	Yes	13	28.79 (27.35)	21.88 (0.0–75.00)	0.215
	No	984	19.44 (20.55)	13.28 (0.0‐–93.75)	

Abbreviations: CS, cesarean section; PLTCs, potentially life‐threatening maternal conditions; SD, standard deviation; SVD, spontaneous vaginal delivery.

^a^ By Mann–Whitney *U* test (for variables with 2 categories) or Kruskal‐Wallis test (for variables with ≥3 categories).

^b^ Significance values have been adjusted by the Bonferroni correction for multiple comparisons.

### 3.2 The impact of PLTCs exposure on health‐related functional disability

To compare the WHODAS 2.0 score (functional disability) between the two groups at 6 months, a Mann–Whitney U test was performed. The median WHODAS 2.0 score for women with PLTCs (Mdn = 25.00, IQR = 40.04) was significantly higher than that of women without PLTCs (Mdn = 9.38, IQR = 14.84), indicating a greater level of disability in the PLTCs group. Based on the Mann–Whitney *U* test, the results indicated that there were statistically significant differences in WODAS 2.0 scores between the groups, *U* = 64 081, *Z* = −10.77, *P* < 0.001. Effect size (*r*) was calculated using the formula *r* = *Z*/√*N*, which was 0.341, indicating a medium effect. Median scores of six domains were also significantly higher in the PLTCs group as compared with those without PLTCs (*P* < 0.001). In addition, the mean ± standard deviation is presented to compare the results with data from other studies. The exposed group displayed a significant difference in the overall WHODAS 2.0 score compared with the control group (*P* < 0.001) (Table 3).

**TABLE 3:** Mann–Whitney *U* test for the differences in WHODAS 2.0 scores between women with and without PLTCs (*n* = 997).

Domains	Exposed group (*n* = 330)		Non‐exposed group (*n* = 667)		Mann–Whitney test			
	Mean (SD)	Median (IQR)	Mean (SD)	Median (IQR)	*U*	*Z*	*r*	*P* value^a^
Overall domain score	30.61 (24.98)	25 (40.04)	14.10 (15.47)	9.38 (14.84)	64 081	−10.77	0.341	<0.001
Cognition	27.24 (24.63)	25 (42.71)	12.48 (17.12)	8.33 (25)	69 969	−9.58	0.303	<0.001
Mobility	36.50 (25.14)	35 (40)	18.78 (19.60)	10 (20)	64 678	−10.68	0.338	<0.001
Self‐care	25.09 (25.77)	18.75 (38)	9.66 (16.14)	0 (12.50)	70 963	−9.59	0.304	<0.001
Getting along with people	26.18 (27.97)	22.50 (40)	10.27 (16.32)	5 (15)	73 149	−8.97	0.284	<0.001
D5a: Home activities	35.77 (30.43)	25 (50)	17.25 (17.42)	12.50 (25)	70 951	−9.32	0.295	<0.001
D5b: Work activity^b^	37.17 (27.62)	25 (53.13)	23.16 (22.22)	18.75 (19)	7726	−4.54	0.258	<0.001
Participation	32.42 (27.57)	25 (50)	15.44 (17.15)	9.38 (21.88)	68 999	−9.66	0.306	<0.001

Abbreviations: IQR, interquartile range; PLTCs, potentially life‐threatening maternal conditions; SD, standard deviation.

^a^ By nonparametric Mann–Whitney test.

^b^ Total participants 310 (119 PLTCs, 195 without PLTCs).

The non‐parametric ANCOVA test revealed significant differences in the overall WHODAS 2.0 score ranks between the groups at 6 months postpartum, after adjusting for baseline disability levels (*F* [2, 994] = 68.7, *P* < 0.001), with the model explaining 12.2% of the variance (adjusted *R* ^2^ = 0.12). Women with PLTCs revealed significantly higher disability scores compared to those without PLTCs. The non‐parametric ANCOVA was also assessed across the six domains of disability. The results revealed statistically significant differences between the groups in all domains (*P* < 0.001). The domain with the largest impact was mobility (*F* = 64.51, η^2^p = 0.115), while relationships with other people had the smallest impact (*F* = 43.71, η^2^p = 0.081) (Table 4).

**TABLE 4:** The impact of PLTCs on WHODAS 2.0 using non‐parametric ANCOVA (distribution‐free test).

Domains	Groups	Type III sum of squares	df	Mean squares	*F*	*P* value	Partial eta‐squared (η^2^p)
Overall domains	Group	10000336.85	2	5000168.43	68.77	<0.001	0.122
	Error	72275013.15	994	72711.28			
Domain 1 (cognition)	Group	7 286 693.30	2	3643346.65	50.51	<0.001	0.092
	Error	71 700 621.20	994	72133.42			
Domain 2 (mobility)	Group	9 348 388.22	2	4674194.11	64.51	<0.001	0.115
	Error	72 024 170.78	994	72458.92			
Domain 3 (self‐care)	Group	8075090.79	2	4037545.397	59.94	<0.001	0.108
	Error	66961238.71	994	67365.431			
Domain 4 (relationship with people)	Group	6177418.62	2	3088709.31	43.71	<0.001	0.081
	Error	70245149.38	994	70669.16			
Domain 5 (life activities)	Group	7997147.71	2	3998 573.85	55.68	<0.001	0.101
	Error	71383068.28	994	71813.95			
Domain 6 (participation)	Group	8709267.19	2	4354633.59	59.42	<0.001	0.107
	Error	72845493.81	994	73285.21			

Abbreviations: ANCOVA, analysis of covariance; df, degrees of freedom; PLTCs, potentially life‐threatening maternal conditions.

In the first model, which adjusted for baseline WHODAS 2.0 scores and maternal health conditions, PLTCs moderately predicted the 6‐month WHODAS 2.0 score (η^2^p = 0.091, *P* < 0.001), while the baseline WHODAS 2.0 score was weakly statistically significant (η^2^p = 0.006, *P* < 0.016). However, this model explained only 12.2% of the variance, excluding potential confounders such as sociodemographic factors and obstetric history (Table S1).

To address this limitation, we performed the non‐parametric ANCOVA test again, including all covariates, which increased the explanatory power of the variance (*R* ^2^ = 26.6%) and identified important confounders such as rural residence (*F* = 22.03, *P* < 0.001, η^2^p = 0.023), governmental employment (*F* = 22.30, *P* < 0.001, η^2^p = 0.012), cesarean delivery (*F* = 14.26, *P* < 0.001, η^2^p = 0.015), and having a history of stillbirth (*F* = 6.26, *P* = 0.01, η^2^p = 0.006), which had statistically significant effects. After adjustment, the baseline WHODAS 2.0 score became non‐significant (*P* = 0.15), and the effect of maternal health conditions was halved (η^2^p = 0.043). This underscores the need for covariate adjustment to avoid biased estimates in the PLTC study (Table 5).

**TABLE 5:** Comparison of health‐related functional disability between women with and without PLTCs using non‐parametric ANCOVA.

Source		Type III sum of squares	df	Mean square	*F*	*P* value	η^2^p
Corrected model		21846690.74^a^	28	780238.96	12.50	<0.001	0.266
Intercept		1797280.34	1	1797280.34	28.79	<0.001	0.029
WHODAS 2.0 at baseline		130885.03	1	130885.03	2.10	0.148	0.002
Age (≥35 years)		124052.59	1	124052.59	1.99	0.159	0.002
Rural residents		1437799.18	1	1437799.18	23.03	<0.001	0.023
Marital status(unmarried)		75781.00	1	75781.00	1.21	0.27	0.001
Occupation (ref. housewives)	Employed	760415.10	1	760415.10	12.18	0.001	0.012
	Merchant	58418.34	1	58418.34	0.94	0.33	0.001
Education (ref. college and above)	No formal education	6848.65	1	63848.65	1.03	0.31	0.001
	Primary level	3360.62	1	3360.62	0.05	0.82	0.000
	Secondary level	21586.31	1	21586.31	0.35	0.56	0.000
Partner education (ref. college and above)	No formal education	4321.30	1	4321.30	0.07	0.79	0.000
	Primary level	355922.38	1	355922.38	5.70	0.02	0.006
	Secondary level	247819.02	1	247819.02	3.970	0.05	0.004
Wealth index level (ref. middle)	Poor	178945.34	1	178945.34	2.87	0.09	0.003
	Rich	11484.40	1	11484.40	0.184	0.67	0.000
Labor onset (ref. SVD)	Elective CS	13927.96	1	13927.96	0.22	0.64	0.000
	Induced	208500.60	1	208500.60	3.34	0.07	0.003
Mode of delivery (ref. SVD)	Instrumental	1030529.14	1	1030529.14	16.51	<0.001	0.017
	CS	889861.56	1	889861.56	14.26	<0.001	0.015
Iron folate intake (ref. with compliance)	Never taken	940799.79	1	940799.79	15.07	<0.001	0.015
	Without compliance	2272078.65	1	2272078.65	36.40	<0.001	0.036
Alcohol intake during pregnancy		4570.95	1	4570.95	0.07	0.79	0.000
Parity ≥5		40045.95	1	40045.95	0.64	0.42	0.001
Twin childbirth		90087.05	1	90087.05	1.44	0.23	0.001
Having history of still birth		390905.23	1	390905.23	6.26	0.01	0.006
Late ANC booking and/or incomplete visits		1011292.23	1	1011292.23	16.20	<0.001	0.016
Having chronic hypertension		735378.97	1	735378.97	11.78	<0.001	0.012
Had malaria infection during pregnancy		595.11	1	595.11	0.01	0.92	0.000
Maternal health status (PLTCs)		2 745 697	1	2745697.01	43.98	<0.001	0.043
Error		60428659.3	968	62426.30			
Total		330 529 347	997				
Corrected total		82 275 350	996				

Abbreviations: ANCOVA, analysis of covariance; CS, cesarean section; df, degrees of freedom; PLTCs, potentially life‐threatening maternal conditions; SVD, spontaneous vaginal delivery.

^a^ *R* squared = 0.266 (adjusted *R* squared = 0.244).

## 4 DISCUSSION

We investigated PLTCs, including hemorrhagic disorders, hypertensive disorders, other systemic disorders, and severe management indicators^2^ and described their effects on health‐related functioning and disability during an extended postpartum period compared to that of women without PLTCs. Assessing health‐related functioning and disability is a relatively recent issue, and the WHODAS 2.0 represents a new item for assessing this domain.^26^ To our knowledge, limited studies have been conducted on the WHODAS specifically among the childbirth women, with no reports on long‐term postpartum. Existing studies have predominantly used the retrospective design^7^, ^9^, ^27^ or the 12‐item version of WHODAS 2.0^10^, ^28^ which explains only 81% of the 36‐item version.^5^

We found that women with PLTCs had significantly higher disability (WHODAS 2.0 scores) in the overall domain compared to those without, indicating more disability. The effect size was medium (0.341). Factors like lower education, rural residence, governmental employment, CS delivery, prenatal care use, history of stillbirth, and chronic hypertension were associated with higher disability. When adjusting for covariates, the model increased the powers of variance explained, indicating these factors' confounding effects.

Partners with lower educational levels had significantly higher WHODAS 2.0 scores, suggesting a relationship between lower educational levels and poor health outcomes.^29^ This is supported by findings that relate education to maternal health care‐seeking behavior.^30^ Chronic hypertension was strongly associated with increased WHODAS 2.0 scores. Hypertension during pregnancy frequently leads to complications like pre‐eclampsia, which can affect physical functioning and mental health postpartum. Research indicated that hypertension is a key driver of disability, affecting domains such as cognitive impairment and functional disability.^31^ Lack of iron‐folate intake was associated with a higher WHODAS 2.0 score. Anemia is a risk factor for reducing physical activity and fatigue, which may explain the elevated results. This is consistent with a study on nutritional intervention for mitigating morbidity and disability.^32^ Anemia and chronic hypertension management during pregnancy should be focused on in antenatal care (ANC) to mitigate long‐term functioning disabilities. The risk could be reduced by integrating a nutritional supplementation program in routine prenatal care visits.^33^ Late ANC booking or incomplete visits were associated with increased WHODAS 2.0 scores. Late prenatal care booking and incomplete recommended visits may lead to delayed diagnosis and management of maternal health conditions such as anemia, infection, and hypertension, which are significantly associated with functional disability and cognitive impairment.^31^, ^34^

The history of stillbirth is associated with elevated WHODAS 2.0 scores, which highlights the psychological impact of such experiences on women's health. This is in line with a study that indicated women who have experienced stillbirth may face long‐term psychological stress and increased risk of mental health problems.^35^ Cesarean delivery was associated with a higher WHODAS 2.0 score, which highlights the influence of the outcome on long‐term women's health. This is in line with an earlier study that indicated cesarean deliveries could result in long‐term recovery time and delayed physical activity resuming after giving birth.^36^, ^37^ While CS is often mandatory in high‐risk pregnancies, it is linked with postpartum pain, psychological distress, and limited mobility, which are key drivers of functional limitation.^38^

The result of this study indicates the significant difference in disability between women who faced PLTCs and those who did not at 6 months postpartum with median scores of 25.00 versus 9.38. This difference was significant (*U* = 64 081, *Z* = −10.77, *P* < 0.001), with a medium effect size of (*r* = 0.34). This result aligns with a previous study indicating that obstetric complications can lead to long‐term functional impairments.^39^ In contrast, other studies have reported no differences in disability level between the groups in the postpartum period. These inconsistencies may come from differences in demographic factors or sample size.^10^

Our study also indicated that there were significant differences between the groups at 6 months postpartum, even after adjusting for the baseline WHODAS score in the non‐parametric ANCOVA test (η^2^p = 0.091, *P* < 0.001). This result is in line with a study that links maternal morbidity to role limitations.^10^ However, the initial model explained only 12.2% of the variance, suggesting that PLTCs do not fully explain the disability outcome. In addition, women with PLTCs had higher disability scores across all the domains. Mobility was the most affected domain with the effect size (η^2^p = 0.115), likely reflecting physical consequences of PLTCs such as prolonged recovery after maternal hemorrhage or cesarean delivery.^28^ In contrast, the relationships with people domain (η^2^p = 0.081) showed the smallest impact, which is in line with the study that found relationships with people and self‐care are the least affected domains.^9^

When sociodemographic and obstetric covariates were included, the model's explanatory power doubled (*R* ^2^ = 26.6%), indicating their significant contributions. Significant predictors were living in a rural area, being government employed, inadequate prenatal care, cesarean delivery, incomplete iron‐folate intake, having a history of stillbirth, and having chronic hypertension (*P* < 0.001), which is consistent with studies showing that marginalized populations experienced more difficult postpartum situations.^36^, ^37^, ^40^, ^41^

After adjusting, baseline disability (WHODAS 2.0 score) was no longer significant (*P* = 0.15), suggesting its initial significance was confounded by systemic inequalities such as access to health care rather than innate health status, which was the cause of its initial significance. Screening for risk factors such as lower educational status, rural residence, lack of on‐time ANC booking, and not taking iron‐folate supplementation during prenatal care could identify women at risk for postpartum disability. Unmeasured variables, such as social support and mental health, are indicated by the moderate explained variation (26.6%). Qualitative approaches should be used in future research to examine contextual or cultural mediators. Targeted treatments are necessary to support women with PLTCs, as evidenced by their elevated WHODAS scores. It is important for healthcare providers to understand that these women might need extra support networks and resources in order to properly manage their functional limitations. Moreover, the statistically significant differences observed in all the specific domains, such as self‐care and mobility, suggest that tailored rehabilitation services focusing on these areas could improve recovery for affected women. Future research should consider trials targeting high‐impact domains such as mobility. Health care providers are advised to consider the long‐term functioning disability of PLTCs women in addition to the existing standard physical examination.

The strengths of this study are the use of rank‐based ANCOVA to address non‐normality, sequential modeling to isolate confounders, and domain‐specific analysis, enabling targeted interventions. Although the study offers insightful information, it is important to recognize its limits. The use of self‐reported measures may introduce bias, as participants might overreport or underreport their disabilities due to personal perceptions or social stigma. Factors such as history of mental health, social support, or cultural practices were not assessed but may affect functioning. This study is conducted in hospitals, which is an unavoidable setting because the criteria for PLTCs would be required for accurate identification that is difficult to be verified in the community context. Similarly, women without PLTCs (non‐exposed group) were also recruited from health facilities to make sure that these women clinically did not have PLTCs. The other limitation of this study is unmatching among the exposed and nonexposed groups.

## 5 CONCLUSION

This study identified significant sociodemographic and clinical disparities between women with and without PLTCs, including employment status, partners with low educational levels, rural residence, incomplete antenatal care engagement, cesarean delivery, history of stillbirth, and non‐compliance with iron‐folate supplementation. These factors are not only to increase the risk of PLTCs but also to increase mothers' functional disability, which may be due to prolonged recovery, limited medical services access, and untreated health complications. This study underscores the significant impact of PLTCs on women's functioning ability in long‐term postpartum. Prioritizing equity‐focused post‐natal care and integrating disability assessments in the post‐natal care programs could enhance long‐term outcomes for PLTC survivors.

## AUTHOR CONTRIBUTIONS

FTB designed the study, managed the analyses, and wrote the draft of the manuscript. GFA, YBZ, and ABK were involved in designing and reviewing the manuscript. All authors have read and approved the final manuscript.

## FUNDING INFORMATION

No particular funding was given for this research.

## CONFLICT OF INTEREST STATEMENT

The authors declare that they have no competing interests.

## Supporting information


Table S1.

## Data Availability

When a reasonable request is made, the corresponding author can provide the datasets used and/or analyzed in this study.
