# Supplementary material for: Functional status of women with and without potentially life‐threatening maternal conditions after 6 months postpartum: A cohort study
Source: Int J Gynaecol Obstet. 2026 Mar 13;174(3):1338–49. doi: 10.1002/ijgo.70905 (PMC13505273; doi:10.1002/ijgo.70905)
Supplement: Supplementary file 1 [file IJGO-174-1338-s001.docx]

Table S1: The effect of PLTCs on WHODAS 2.0 score using Non-parametric ANCOVA

| Source | Type III Sum of Squares | df | Mean Square | F-value | P-value | Partial Eta Squared |
| --- | --- | --- | --- | --- | --- | --- |
| Corrected Model | 10000336.850^a^ | 2 | 5000168.43 | 68.77 | <0.001 | 0.122 |
| Intercept | 41700051.230 | 1 | 41700051.23 | 573.50 | <0.001 | 0.366 |
| Baseline WHODAS 2.0 score | 426425.235 | 1 | 426425.24 | 5.87 | 0.016 | 0.006 |
| Maternal Health Status (PLTCs) | 7208709.144 | 1 | 7208709.14 | 99.14 | <0.001 | 0.091 |
| Error | 72275013.150 | 994 | 72711.28 |  |  |  |
| Total | 330529347.000 | 997 |  |  |  |  |
| Corrected Total | 82275350.000 | 996 |  |  |  |  |
| a. R Squared = .122 (Adjusted R Squared = .120) | | | | | | |
